# Community health workers: working conditions and occupational health

**DOI:** 10.47626/1679-4435-2021-622

**Published:** 2021-12-30

**Authors:** José Nilton dos Santos Ferreira, Renata Lívia Silva Fonseca Moreira de Medeiros, Yuri Charllub Pereira Bezerra, Geane Silva Oliveira, Ocilma Barros de-Quental

**Affiliations:** 1 Departamento de Enfermagem, Faculdade Santa Maria, Cajazeiras, PB, Brazil.

**Keywords:** community health workers, employee health, occupational health, agentes comunitários de saúde, saúde do trabalhador, saúde ocupacional

## Abstract

**Introduction:**

Community health workers are qualified professionals who provide health services that are culturally appropriate for a region. In their work routine, these professionals experience the most diverse and risky situations, which may cause vulnerability to the process of illness and even compromise care to users of the Brazilian Unified Health System. Therefore, understanding the factors associated with the work process that imply the illness of community health workers helps increase the safety of the service provided by these workers.

**Objectives:**

To analyze the implications for illness arising from the work process of community health workers.

**Methods:**

A cross-sectional, descriptive, quantitative study was conducted with the participation of all community health workers in the municipality of Baixio, state of Ceará, Brazil. The interviews were conducted by means of a questionnaire containing questions to assess the work context with quantitative answers, from February to April 2019. Data were analyzed by the Shapiro-Wilk normality test, followed by Spearman correlation test. Differences were considered significant at a p-value < 0.05.

**Results:**

High demand for results in the workplace and insufficient work instruments demonstrated a directly proportional correlation with headache, body pain, back pain, and vision disorders (p < 0.05).

**Conclusions:**

Working conditions can directly affect the health of community health workers. Therefore, public strategies are essential to ensure workers’ safety and quality of care for users of the Brazilian Unified Health System services.

## Resumo

**Introdução:**

Agentes comunitários de saúde são profissionais habilitados que fornecem serviços de saúde culturalmente adequados a uma região. Em suas rotinas laborais, esses profissionais enfrentam diversas e arriscadas situações, que podem ocasionar vulnerabilidade frente ao processo de adoecimento e, até mesmo, comprometer o atendimento dos usuários do Sistema Único de Saúde. Por isso, compreender os fatores associados ao processo de trabalho que implicam o adoecimento dos agentes comunitários de saúde auxilia no aumento da segurança para a prestação de serviços desses trabalhadores.

**Objetivos:**

Analisar as implicações para o adoecimento decorrentes do processo de trabalho dos agentes comunitários de saúde.

**Métodos:**

Foi realizado um estudo transversal, descritivo e quantitativo com a participação de todos os agentes comunitários de saúde do município de Baixio, Ceará. As entrevistas foram realizadas por meio de questionário contendo questões de avaliação do contexto de trabalho, com respostas quantitativas, entre os meses de fevereiro e abril de 2019. Os dados foram analisados pelo teste de normalidade Shapiro-Wilk, seguido pelo teste de correlação de Spearman. As diferenças foram consideradas significativas com valor de p < 0,05.

**Resultados:**

A forte cobrança por resultados no ambiente de trabalho e os instrumentos insuficientes de trabalho demonstraram uma correlação diretamente proporcional com dor de cabeça, dor no corpo, dor nas costas e distúrbios da visão (p < 0,05).

**Conclusões:**

As condições de trabalho podem afetar diretamente a saúde dos agentes comunitários de saúde. Assim, são fundamentais estratégias públicas que garantam a segurança do trabalhador e a qualidade do atendimento dos usuários dos serviços do Sistema Único de Saúde.

## INTRODUCTION

Community health workers are qualified professionals who provide health services that are culturally appropriate for a region. They have a deep understanding on the culture and language of the community and play crucial roles for the increase in the coverage of basic health services.^1^ In Brazil, this profession is regulated by Laws no. 11,350 (2006) and no. 13,595 (2018), which recognize this category as health care professionals, making them responsible for activities aimed at health promotion and disease prevention, through home or community actions, either individual or collective, developed according to the guidelines of the Brazilian Unified Health System (Sistema Único de Saúde, SUS).^2,3^

In the workplace, these professionals are inserted into a multiprofessional team, and, jointly, integrate the family health team, whose area of work covers a delimited territory, where they develop their actions with a determined number of families and people.^4^ For community health workers to successfully perform the interconnection of community and health services with the population, it is essential for them to have quality of life and of work.^5^ However, these workers are susceptible to conditions that negatively affect their physical and emotional health. Furthermore, these individuals experience peculiar work dynamics, i.e., they live and work in the same community, which may lead to additional pressure and overload.^6^

Therefore, considering the vulnerabilities resulting from the work process of community health workers, studies that describe the occupational health scenario of this category are crucial to substantiate public strategies to support worker’s and community health. Hence, the aim of this study was to analyze the implications for illness, resulting from the work process of community health workers. Thus, it may contribute with scientific knowledge that favors safety and health in the daily routine of these professionals, in order for them to provide better health care to the population who use the SUS.

## METHODS

A cross-sectional, descriptive, quantitative study was conducted. The study population consisted of community health workers (n = 18) in the municipality of Baixio, state of Ceará, Brazil. The state included all community health workers who were registered as active workers on the National Registry of Health Establishments (Cadastro Nacional de Estabelecimentos de Saúde) and living in the aforementioned municipality. Retired professionals not working in the business were excluded from the study.

Data were collected through interviews with a semi-structured questionnaire containing questions with quantitative answers on work and risks of illness. The interviews were conducted during meetings of Family Health Teams, at Basic Health Units, and/or at the respondent’s household, from February to April 2019. Participants were informed on research objectives, and, after accepting to participate by signing the Free Inform Consent, the interview was initiated. The study was approved by the Research Ethics Committee of Faculdade Santa Maria (opinion no. 3.258.059).

Data were analyzed using the BioEstat^®^ statistical software, version 5.0 (Instituto Mamirauá, Belém, Brazil). Results were shown in figures or tables, created in the Microsoft Excel^®^ software, version 1808. For categorical variables, absolute and relative frequencies were used. Numerical variables were analyzed through the Shapiro-Wilk normality test, followed by the Spearman correlation test. Variables were considered significant at a p-value < 0.05.

## RESULTS

### CHARACTERIZATION OF COMMUNITY HEALTH WORKERS AND ASSESSMENT OF WORK CONTEXT

The main characteristics of community health workers were described in Table 1.

**Table 1:** Characterization of community health workers participating in the research

Parameters	n = 18
Age, years - median (min.-max.)	45 (25-57)
Gender	
Female	12 (66.7)
Male	6 (33.3)
Educational level	
Complete high school	15 (83.3)
Incomplete higher education	1 (5.6)
Complete higher education	2 (11.1)
Marital status	
Single	2 (11.1)
Married/common law marriage	16 (88,9)
Time of service, years	
Median (min.-max.)	24 (3-27)

Quantitative variables were described as median, followed by minimum (min.) and maximum (max.) values. Categorical variables were expressed as absolute and relative (%) frequency.

Community health workers participating in the study had a mean age of 45 years, were mostly women (66.7%), had complete high school (83.3%), and were married (88.9%); additionally, median time of service was 24 years, with a minimum of 3 years and a maximum of 27 years (Table 1).

The way community health workers assessed their work context was described in Table 2.

**Table 2:** Distribution of community health workers according to work context assessment

Parameter	Work context assessment n = 18		
	Never/seldom n (%)	Sometimes n (%)	Often/always n (%)
Excessive work rhythm	3 (16.7)	14 (77.7)	1 (5.6)
Tasks completed under deadline pressure	2 (11.1)	16 (88.9)	-
High demand for results	6 (33.3)	4 (22.2)	8 (44.5)
Strict rules for the performance of tasks	12 (66.7)	6 (33.3)	-
Insufficient number of people to perform the tasks	17 (94.4)	-	1 (5.6)
Repetitive tasks	1 (5.6)	11 (61.1)	6 (33.3)
Lack of rest breaks during work	7 (38.9)	9 (50.0)	2 (11.1)
Employees excluded from decisions	14 (87.7)	3 (16.7)	1 (5.6)
Communication difficulties between managers and subordinates	16 (88.9)	2 (11.1)	-
Lack of integration in the workplace	11 (61.1)	7 (38.9)	-
Lack of support from managers for professional development	13 (72.2)	5 (27.8)	-

Variables were described as absolute and relative frequency.

With regard to most frequently cited parameters of work context assessment, it was observed that most respondents reported that there was often or always high demand for results (44.5%). Respondents also reported that sometimes the work rhythm was excessive (77.7%), tasks were completed under deadline pressure (88.9%), tasks were repetitive (61.1%), and there was lack of rest break during work (50%) (Table 2). For the remaining variables, most respondents answered that the situations occurred seldom or never.

The way community health workers assessed their work context according to the performance of tasks and associated physical structures was described in Table 3.

**Table 3:** Work context assessment according to the performance of tasks and associated physical structures

Parameters	Work context assessment n = 18		
	Never/seldom n (%)	Sometimes n (%)	Often/always n (%)
Difficult access to information necessary to perform the tasks	17 (94.4)	1 (5.6)	-
Precarious working conditions	14 (77.8)	4 (22.2)	-
Uncomfortable physical environment	14 (77.7)	3 (16.7)	1 (5.6)
Too much noise in the workplace	15 (83.3)	-	3 (16.7)
Insufficient work instruments	8 (44.4)	10 (55.6)	-
Precarious necessary equipment	12 (66.7)	6 (33.3)	-
Inadequate physical space to perform work	16 (88.9)	2 (11.1)	-
Working conditions pose risks to people’s safety	8 (44.4)	8 (44.4)	2 (11.2)
Insufficient consumable supplies	9 (50.0)	9 (50.0)	-

Variables were described as absolute and relative frequency.

In relation to the work context assessment according to the performance of tasks and associated physical structures, the most frequently cited situations were associated with resource limitation. Among respondents, 55.6% said that work instruments were insufficient sometimes, and 50% reported that consumable supplies were insufficient sometimes. With regard to risks, more than a half of participants answered that working conditions posed risks to people’s safety, of which 44.4% said that it occurred sometimes, and 11.1% reported that it occurred often or always. For the remaining variables, most respondents answered that the situation occurred seldom or never (Table 3).

### HEALTH-DISEASE PROCESS AND CORRELATION OF WORKING CONDITIONS OF COMMUNITY HEALTH WORKERS

**Figure 1:**
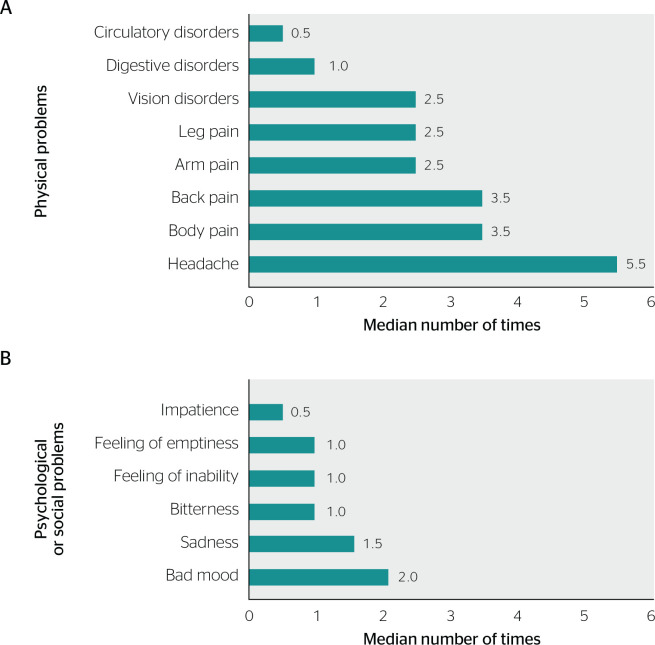
Median number of times, on a scale from 0 to 6, when the reported physical, psychological, and social problems were considered to be essentially caused by work over a 6-month period. Variables were described as the median number of times when the event occurred in the last 6 months, on a scale from 0 to 6 times or more, with Figure 1A showing physical problems, and Figure 1B showing psychological and/or social problems.

The frequency of physical, psychological, and/or social problems essentially attributed to work in the last 6 months was described in Figure 1.

Considering the scale from 0 to 6 times or more, headache was the most frequently reported disorder: the median frequency of work-related headache in the last 6 months was 5.5 times, followed by body pain and back pain (3.5 times). Leg pain, arm pain, and vision disorders had a frequency of 2.5 times during the aforementioned period (Figure 1A). The factors associated with psychological and/or social disorders that occurred most frequently in respondents’ lives over the last 6 months were bad mood (twice) and sadness (1.5 time) (Figure 1B).

**Figure 2:**
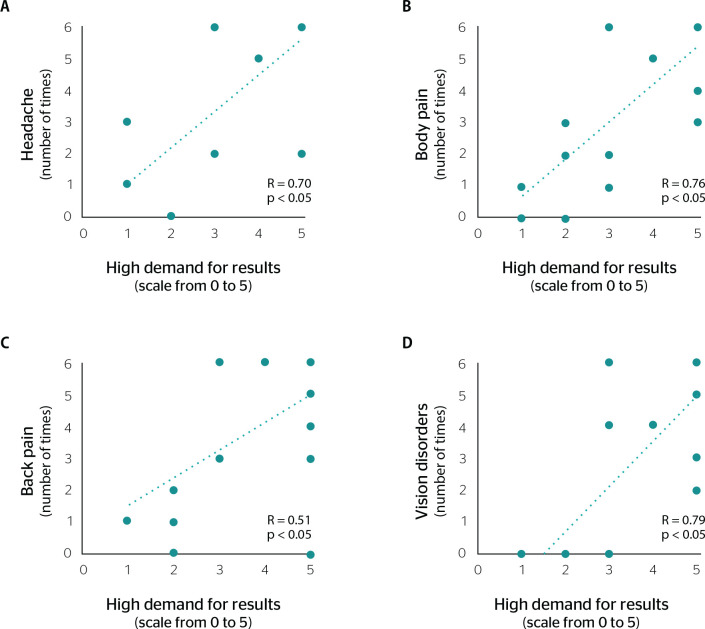
Correlation between high demand for results and associated physical disorders. Headache (A), body pain (B), back pain (C), and vision disorders (D). Statistically significant differences were assessed using the Spearman correlation test, p. R = statistical correlation.

**Figure 3:**
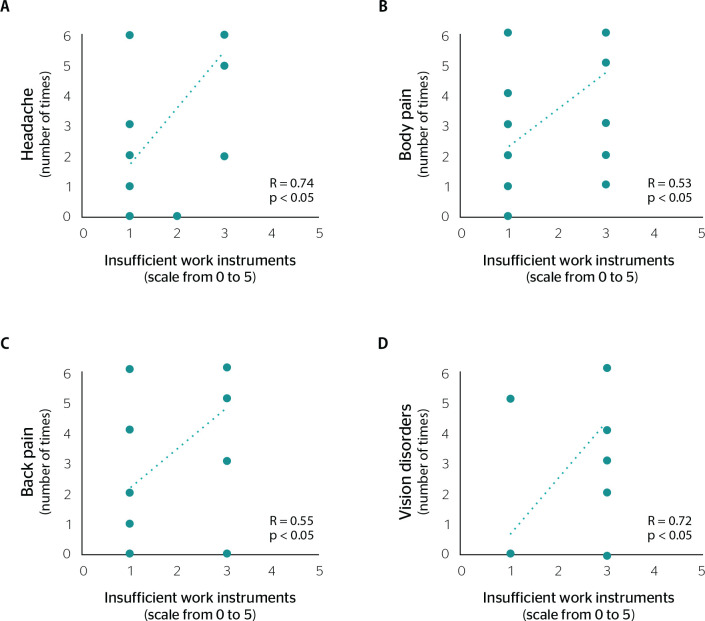
Correlation between insufficient work instruments and associated physical disorders. Headache (A), body pain (B), back pain (C), and vision disorders (D). Statistically significant differences were assessed using the Spearman correlation test, p. R = statistical correlation.

Headache, body pain, back pain, and vision disorders showed a positive correlation with the items related to the work context (Figure 2 and Figure 3).

It was observed that high demand for results in the workplace had a directly proportional positive correlation with headache, body pain, back pain, and vision disorders (p < 0.05) (Figure 2).

Insufficient work instruments also showed an association with a possible impact on worker’s health. The assessment of the parameter ‘insufficient work instruments’ was directly correlated with the number of times reported by health workers for headache, body pain, back pain, and vision disorders (p < 0.05) (Figure 3).

## DISCUSSION

Illness in the workplace is a dynamic process, influenced by establish working conditions and relationships, in addition to the social context in which the worker is inserted. In negative contexts, work may become a causative agent of suffering and physical and mental disease.^7^

In the occupational domain of community health workers, many factors of the work context, such as high demands to achieve goals and lack of system resoluteness and work recognition, contribute to the physical and psychological suffering of community health workers.^8^ In the present study, factors associated with high demand to achieve goals were cited as present in the workplace of community health workers, such as demand for results, excessive work rhythm, deadline pressure, repetitive tasks, and lack of rest breaks. In contrast, workers also reported favorable working conditions, such as good communication between managers and subordinates and support for professional development.

In the scientific literature, it is observed that health workers are often subjected to working that exceed the opening hours of the health unit and invade their private life; to care and/or follow-up of a number of families higher than the proposed; to exposure to unhealthy working conditions; to low pay and lack of social protection; to little work recognition from managers, peers, and users; and to system precariousness that falls on health workers.^9^

Many aforementioned factors reflect the high demands imposed to the work of community health workers, the undervaluing of their work, and the intense emotional involvement with users. These facts together contribute to the development of physical and psychical exhaustion and thus to lack of interest in work.^10^

Community health workers have been increasingly affected by occupational problems that directly interfere with quality of life, such as anxiety, depression, and stress.^11^ In the present study, reports of physical problems were more frequent than those of psychological disorders. Additionally, there was a statistically significant direct correlation between headache, body pain, back pain, and vision disorders with high demand for results in the workplace and with insufficient work instruments.

The high demands experienced by community health workers in their daily routine, both from the public management and from the community, lead to health problems such as stress, anxiety, and many other diseases.^6^ Furthermore, inadequate work environment and precarious work equipment are considered physical loads in the work of community health workers. Factors such as scarcity of work equipment, lack of uniform, and lack of sunscreen and of bags to carry work materials end up leading to physical problems.^12^

Health problems such as musculoskeletal pain are increasingly more frequent among community health workers and may result from working conditions.^13^ Long daily walks, exposure to the sun, and backpack weight end up leading to spinal pain, back pain, leg pain, in addition to causing the worsening and onset of varicose veins and headache.^14,15^ Moreover, there is a high physical demand to perform this type of work, especially for professionals who work in rural areas. They need to make long journeys during home visits and are exposed to animal attacks and climate hazards.^15^

Another important point is lack of equipment and/or instruments to make daily work more efficient, which may pose risks for workers’ health. Therefore, the use of personal safety equipment, the adoption of strategies that contribute with accident prevention, and public policies that promote the health of these workers are essential for the their health.^16^ Furthermore, it is necessary to develop nationwide epidemiological studies that reinforce the risk factors for the health of community health workers, so that these professionals may enjoy a good health status and be able to take care of population’s health.

## CONCLUSIONS

The work process of community health workers may lead to health implications for this group of workers. High demand for results in the workplace and insufficient work instruments show a direct correlation with headache, body pain, back pain, and vision disorders. In order to ensure the physical and emotional integrity of these workers, it is necessary to increase investments on consumable supplies, especially on essential items used by professionals during work journeys and patient care. Moreover, the provision of health care professionals’ qualification focused on the human resource management would be essential for the development and implementation of new strategies to ensure quality of care to users of the SUS, with community health workers exhibiting an excellent performance, with no direct excessive demands for results and negative impacts on worker’s health.
